# Technology adoption review for ageing well: analysis of technical solutions

**DOI:** 10.3389/fpubh.2023.1169192

**Published:** 2023-09-20

**Authors:** Ishaya Gambo, M. Victoria Bueno-Delgado, Kerli Mooses, Francisco J. Melero Muñoz, Rina Zviel-Girshin, Aliaksei Andrushevich, Michael Mrissa, Agnieszka Landowska, Kuldar Taveter

**Affiliations:** ^1^Institute of Computer Science, University of Tartu, Tartu, Estonia; ^2^Department of Computer Science, Obafemi Awolowo University, Ile-Ife, Nigeria; ^3^Department of Information and Communication Technologies, Universidad Politécnica de Cartagena, Antiguo Cuartel de Antigones, Cartagena, Spain; ^4^Technical Research Centre of Furniture and Wood of the Region of Murcia, Yecla, Spain; ^5^School of Engineering, Ruppin Academic Center, Emek Hefer, Israel; ^6^HomeLab, Lucerne University of Applied Sciences and Arts, Lucerne, Switzerland; ^7^Faculty of Applied Mathematics and Computer Science, Belarusian State University, Minsk, Belarus; ^8^InnoRenew CoE, Izola, Slovenia; ^9^Faculty of Mathematics, Natural Sciences and Information Technologies (FAMNIT), University of Primorska, Koper, Slovenia; ^10^Department of Software Engineering, Faculty of Electronics, Telecommunications and Informatics, Gdańsk University of Technology, Gdańsk, Poland

**Keywords:** technology adoption, older adults, ageing well, healthy lifestyle, Internet of Things, Information and Communication Technologies, Quality of Life

## Abstract

While several technological solutions are available for older adults to improve their wellbeing and quality of life, little is known about the gaps between the needs, provided solutions, and their adoption from a more pragmatic perspective. This paper reports on reviewing existing technological solutions for older adults, which span the work life, life in the community, and wellbeing at home. We analyzed 50 different solutions to uncover both negative and positive features of these solutions from the perspective of the impact of technology adoption on the quality of life of older adults. Our approach harnesses holistic reasoning to determine the most suitable technologies available today and provides suggestions for improvement toward designing and implementing better solutions.

## 1. Introduction

Recently, technological solutions have been discovered to cater for the challenges associated with the ageing population (1). Consequently, technology adoption is increasingly important in public health intervention programs to improve the older population's Quality of Life (QoL). Indeed, both mature and evolving technologies have been successfully applied for improving the QoL and health of older adults (2–4).

However, despite the proliferation of technological solutions, and their inherent benefits in improving the QoL of older adults, there seems to be a considerable gap in terms of technology adoption. On one hand, the low level of adoption of new technologies in the ageing population is due to the frequent reluctance and lack of motivation of older adults, and insufficient support available to them (5, 6). On the other hand, the low level of adoption rate is connected to the lack of compliance with policies and strategic frameworks of healthcare and wellbeing. Besides, other complexities associated with multiple factors, such as lack of awareness of potential impact and prevailing bureaucracy, have had a negative impact on technology adoption (1, 7, 8). In addition, there is a lack of quality studies of technology adoption (9–11).

From a more technical perspective, the inability to meet older adults' requirements, including emotional requirements (12), in the design of the technological solutions could endanger human lives (13), which eventually also leads to a low level of adoption. Remarkably, several researchers have pointed out that if the needs of older adults are not adequately captured in the analysis and design process, the system's functionality will not be trusted and reliable (14–16).

The overarching goal of this paper is to analyze the existing technical solutions, as contained in the publications, projects, and patents. In the paper, we answered two important research questions: (i) What are the gaps reported in the literature between the needs, production, and adoption of technology for ageing? and (ii) what are the prevalent challenges associated with technology adoption?

This paper analyzes a selection of recent and most relevant technical solutions from the technology adoption perspective. The technologies have been selected as part of the SHELDON COST action number CA16226 based on a review of existing work by the members of the action. Due to the very large amount of activity in this domain, it does not constitute an exhaustive overview, however it provides a selection of the current most relevant initiatives identified by the members of the COST action. Firstly, our analysis unveils both negative and positive aspects of technology adoption on QoL of older adults. Secondly, we further evaluate the results of the analysis by means of statistical measures. Thirdly, the main contribution of this paper are the recommendations and policy implications we can deduce from the analysis. Clearly, the focus is to understand the gaps between the needs, production, and adoption of solutions targeted at older adults.

The structure of the paper is as follows. Section 2 describes the background and motivation, while Section 3 discusses the theoretical frameworks used as guidelines for analyzing the technical solutions. This is followed by the description of our methodological approach in Section 4. Section 5 contains the analysis of the results and Section 6 presents the discussions. Section 7 presents the strengths and limitations of the study. Finally, Section 8 concludes the paper with some recommendations and suggestions.

## 2. Background and motivation

There is a rapid growth in the number of older adults across all continents. According to Ollevier et al., (17), the expected growth of the number of older adults would be well above 60% in another 15 years. According to the reports on the ageing of the world population by the United Nations (18, 19), by 2030, ~1 billion older adults will make up 12% of the whole world population (20). In this context, a number of challenging problems will have to be solved because of the ageing population (21).

Technology has a great potential in providing the support needed for enhancing the healthy lifestyle of older adults (22–24). The development and adoption of this kind of technology is a crucial factor for older adults to compensate for psychological, social, and biological changes occurring in them over time (2), such as the loss of adaptability and functional impairments (25).

Interestingly, the technologies available for older adults have engendered significant changes in recent times. Notably, we have witnessed the use of assistive technologies (22, 26, 27), health monitoring systems, the Internet of Things (IoT) solutions (e.g., wearable devices) (23, 28, 29), smart sensors (3, 30), medication reminders (31), telemedicine applications (32), and social networking applications (9) for enhancing the QoL of older adults.

It is noteworthy that the Ambient Assisted Living (AAL) and Enhanced Living Environments (ELE) technologies comprise significant contributions from researchers in ICT and psychology (33–35). Remarkably, the idea of an ELE refers to the ICT-related part of AAL, which means that ELEs incorporate all ICT advancements to assist AAL (36). The psychological aspects of AAL deal with human behaviors, affects, emotions, and desires. On the other hand, ELE focuses on designing and implementing suitable technologies based on psychological theories of automated systems (37). Additionally, ELE incorporates the most recent innovative achievements in IoT to create better ICT solutions for improving the health and wellbeing of older adults (36).

Encouraging older adults to adopt the technologies aimed to improve their health and wellbeing has received a lot of attention in the research literature (38–40). It is worth of mentioning that technology is repeatedly mentioned to support ageing in place (41, 42). For example, in the FeelGood project (43), a framework was created for supporting and promoting self-management of wellbeing concerns through technology adoption. Also, technological innovations in the Netherlands have enabled to increase the number of dwelling places suitable for older adults (44). However, while most organizations are optimistic about the impact of technology on improving healthy lifestyles of older adults, they tend to focus on its cost rather than its benefits (45–49).

Although technological innovation promises to continuously enhance the health and wellbeing of older adults, the seamless adoption of such technologies from both the human and technical perspectives can be a limiting factor for a sustainable breakthrough or progress (50, 51). Therefore, there is a need to identify and investigate existing solutions and analyze them for their strengths and weaknesses from the technology adoption perspective.

## 3. Theoretical framework

Although the terms “technology acceptance” and “technology adoption” are sometimes used interchangeably, they are not synonymous. On the one hand, “technology acceptance” is a perception of technology that is impacted by various factors. These factors include frequency of use, usage experience, ease of use, usefulness, attitude, usage knowledge and enjoyment (52). On the other hand, “technology adoption” is a process that starts with knowledge of the technology and ends with acceptance and full utilization of the technology. Accepting technology without adopting it is therefore conceivable, but full adoption is impossible without acceptance (53). In the literature, several technology adoption theories exist. However, this paper examines technology adoption among older adults using two theoretical frameworks. The first is the Technology Acceptance Model (TAM) by Davis (54), and the second is Everett Rogers' Diffusion of Innovation (DOI) theory (55).

### 3.1. TAM

TAM is a widely used adoption theory (56–58) that focuses on how people make technology adoption decisions (59, 60). It was derived from the Theory of Reasoned Action (TRA), which is a socio-psychological theory that determines how people will act given their preceding attitudes and Behavioral Intentions (BI) (61, 62). BI is “the degree to which an individual has formulated conscious plans to perform or not to perform some specified behavior in the future (54).” It is predicted by both attitude and perceived usefulness (53).

Further, TAM was formulated to predict and explain technology acceptance and use. In this regard, Davis et al. (59) proposed two significant factors as critical determinants of technology adoption: Perceived Usefulness (PU) and Perceived Ease of Use (PEOU). According to Davis (54), Perceived Usefulness is “the degree to which a person believes that using a particular system would enhance his or her job performance,” while Perceived Ease Of Use is “the degree to which a person believes that using a particular system would be free of effort.” Thus, PU and PEOU are technological variables that emphasize people's attitudes, perceptions, and interactions with technology (63).

Over the years, this area has further developed and TAM has been extended. One such extension is TAM 2, which replaced the attitudinal component of TAM with a social element termed as Subjective Norm (SN) (64). The theory behind TAM 2 claims that cognitive instrumental processes explain perceived utility and usage intentions (e.g., job relevance, output quality, outcome demonstrability, and perceived simplicity of use) as well as social influence processes (subjective norm, voluntariness, and image) (64). Another extension of standard TAM is the Unified Theory of Acceptance and Use of Technology (UTAUT). UTAUT differs from TAM in that it includes social and environmental variables and technological factors as determinants of behavioral intention (65).

Notably, many studies have shown that a strong intention of technology usage results in a high probability of actual usage (66). This implies that a person intending to use technology will most likely use it. As a foundational technology adoption theory, TAM is useful in examining the potential adoption of a given technology among older adults. Yap et al. (67) divided the antecedents of technology usage among older adults into the following seven categories: technological, psychological, social, behavioral, cost-related, personal, and environmental. Antecedent, in this case, indicates pre-existing factors that determine or influence technology adoption by older adults. The source (67) reviewed twenty-six (26) research articles on technology adoption, most of them focusing on TAM and its variables PU and PEOU.

### 3.2. DOI

Everett Rogers did put forward the Diffusion of Innovation (DOI) theory for examining technology adoption and determining how technological innovations diffuse within communities (55, 68). On one hand, diffusion is the process by which an innovation spreads over time and through specific channels among the people within a social system (69, 70). On the other hand, innovation is “an idea, practice, or object perceived as new by an individual or other unit of adoption (69, 70).”

Rogers et al. (69) distinguished between the processes of innovation decisions by individuals and groups. To reduce innovation uncertainty, decision-making units must follow a specific procedure before deciding whether to accept or reject an innovation (69, 70). As observed by Zhang (71), communication channels, innovation attributes, adopter characteristics, time, and the social system are the five essential variables that determine the success of innovation. Regarding technology adoption, the DOI theory recognizes the following five stages: knowledge or awareness, persuasion, decision, implementation, and confirmation.

### 3.3. Inferences observed in TAM and DOI

We observed that TAM is limited as a theory because of its position that technology adoption by older adults solely depends on the features of particular technologies to be adopted. In reality, older adults also think about how a technology enables their lifestyle in ways they value (67). Therefore, TAM does not sufficiently explain the adoption of technologies by older adults. To compensate for that, we have also incorporated the Diffusion of Innovation (DOI) theory to complement TAM for a more holistic analysis. Overall, the growing impact of technology on older adults cannot be underestimated. It has been observed that technology usage improves social, mental, and emotional wellbeing of older adults, while also decreasing their feeling of loneliness (72). Therefore, we used both TAM and DOI as the significant drivers of technology adoption among older adults as has also been reported in Heo et al., Mahoney, Hastall et al., and Cahill et al. (73–76).

Differently, we blended the TAM and DOI theories to provide adequate insight into the analysis of technology adoption among older adults for improving the QoL and facilitating healthy lifestyle. In particular, we used from the TAM model as technological issues the improved wellbeing, ease of use, willingness to accept technology, and understandability. At the same time, we used from the DOI model as technological issues the technological awareness, willingness to accept technology, effectiveness, and understandability. Based on these technological issues identified from the two theories, we formulated hypotheses for statistical analysis.

## 4. Methods

We designed our study using a mixed method consisting of the methods of qualitative explorative approach (77), and quantitative research (78), which have been synthesized as is described by Dixon-Woods et al. (79). The qualitative explorative approach focuses on a critical and extensive review of the existing solutions, while the quantitative research involves using statistical measures to further improve the analysis.

**Figure 1:**
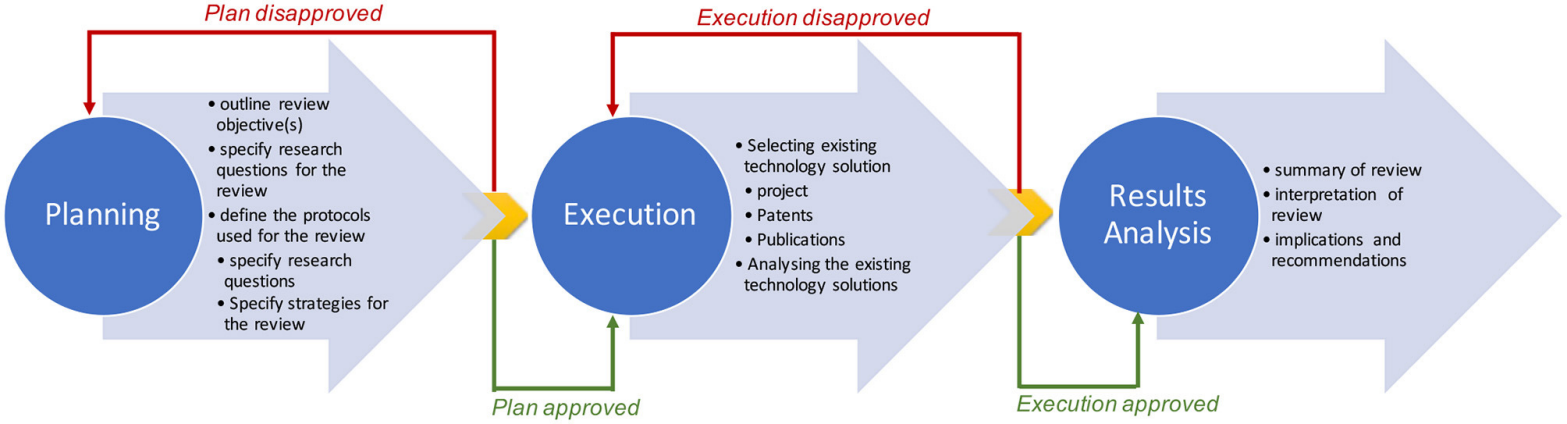
Systematic process for technology adoption review: adapted from Kitchenham (80).

We followed the systematic process described in Figure 1 to analyze the shreds of evidence reported about the existing solutions by answering the research questions mentioned in Section 1. Figure 1 is a modified version of the three-step process of systematic review: *planning, execution (i.e., carrying out the research), and result analysis* (80).

In the “*planning”* phase, we outlined our review objective(s), specified the questions for the review, and defined the protocols used for the review. In the “*execution”* phase, we selected and analyzed the existing technology solutions. Finally, we summarized the review during the “*result analysis”* phase.

Overall, we analyzed 50 existing solutions^1^—projects, patents, and publications—to uncover both negative and positive examples of technology adoption and their impact on QoL of older adults. The selected 50 solutions used for the technology adoption review are part of the SHELDON COST action number CA16226 by the SHELDON Working Group 4.4, which aims to review the current state of the art in technologies for smart living environments. The technologies presented are chosen by the action members based on their relevance and impact in this domain, especially for improving the QoL of older adults. The authors and other COST action members selected the most relevant technologies, projects, patents, and papers for this paper's analysis. We used keyword-based queries in the main online search tools that give a representative sample of the most promising initiatives from the COST action, which include projects, patents, or technological systems.

All the authors were involved in reviewing the 50 existing solutions. Based on the guidelines provided by Kitchenham (80), we adopted the random selection technique to determine the reviewers of particular existing solutions. Consequently, each author decided on the document—project, patent, or technological system—to be reviewed from the list of the documents provided by the SHELDON Working Group 4.4 on technology adoption. The last author coordinated the review process. The objective of the review was 2-fold. The first goal was to understand the gap between the need, solutions, and technology adoption. The second goal was to understand the adoption challenges and the features of the solutions reported in various documents. Based on the resulting reviews, we analyzed from the technology adoption perspective the technical solutions provided by different projects.

### 4.1. Procedure for qualitative analysis of technology adoption

We summarize the reviews provided by the authors by identifying the strengths and weaknesses of each existing solution that was analyzed. For that purpose, three analysis questions were asked for each solution as guides for understanding the gap between the need, solution, and technology adoption. These questions also served as the roadmap for understanding the adoption challenges faced by these solutions that have been reported in various documents. The analysis questions were as follows:

1. AQ1: Has the technology been tried out?
2. AQ2: Has the technology been tried out in a case study involving real end users?
3. AQ3: How well did the end users adopt the solution?

Additionally, we used ten (10) criteria based on the above analysis questions to elaborate our analysis. Table 1 describes the criteria that were applied to the analysis.

**Table 1:** Criteria for analyzing technology adoption.

**SN**	**Criteria used for analysis**	**Key**	**Aspects**
1.	Technology has been used or tried out	+	Positive
2.	Technology has not been used or tried out	−	Negative
3.	End users have started using the technology	+*	Positive
4.	End users have not started using the technology	−*	Negative
5.	Technology has been tried out in a case study by real users	++	Positive
6.	Technology has not been tried out in a case study by real users	–	Negative
7.	Prototype assessment/evaluation with real end users	±	Positive
8.	Prototype assessment/evaluation without real end users - only simulation	≠	Negative
9.	The end users have adopted the technology well	1	Positive
10.	The end users have not adopted the technology well	0	Negative
11.	Information about the items 1–10 is not available	NA	NA

### 4.2. Procedure for quantitative assessment of analysis

We employed a quantitative assessment method to establish the suitability of a particular technology for ageing well. Regarding that, we formulated the null hypothesis *H*_0_ and alternative hypothesis *H*_1_, which were later subjected to a statistical test to verify their validity. In particular, we used the *T*-test and correlation analysis. We tested *H*_0_ with ten (10) randomly selected people within the age range between 60 and 75 by asking them a number of questions based on the TAM and DOI theoretical frameworks.

The structure of the questions that were used to source data from the 10 randomly selected persons is shown in Table 2. The 10 randomly chosen persons are from Tartu, Estonia. Following their verbal consent, they were interviewed informally. As Table 2 reflects, the given technology adoption issues were selected because they have been regarded as the most prevalent ones in the literature (73–76). The questions were designed to capture the individual's opinions on the possible use of a particular technology for improving the QoL of older adults. This is important for determining the levels of technology acceptance of end users for using these technologies.

**Table 2:** The identified technology adoption issues.

**SN**	**Identified issue and the corresponding theory**	**Response**
1.	Technological awareness—DOI	Either: SA, PA, N, PD, SD
2.	Support—DOI and TAM	Either: SA, PA, N, PD, SD
3.	Improve wellbeing—TAM	Either: SA, PA, N, PD, SD
4.	Safety and security—DOI	Either: SA, PA, N, PD, SD
5.	Ease of use—TAM	Either: SA, PA, N, PD, SD
6.	Privacy and confidentiality—DOI	Either: SA, PA, N, PD, SD
7.	Affordability—DOI	Either: SA, PA, N, PD, SD
8.	Confidence and trust—DOI and TAM	Either: SA, PA, N, PD, SD
9.	Willingness to accept technology—DOI and TAM	Either: SA, PA, N, PD, SD
10.	Satisfaction—DOI and TAM	Either: SA, PA, N, PD, SD
11.	Effectiveness—DOI	Either: SA, PA, N, PD, SD
12.	Understandability—DOI and TAM	Either: SA, PA, N, PD, SD

SA, Strongly agree; PA, Partially agree; N, Neutral; PD, Partially disagree; SD, Strongly disagree.

As Table 2 shows, technological awareness is a factor aligned with DOI. It is one of the innovation model's stages of technology adoption. For this study, technological awareness refers to the knowledge of older adults about the existing technology.

Additionally, support focuses on improving wellbeing, safety, security, ease of use, privacy and confidentiality, affordability, confidence, trust, willingness to accept technology, satisfaction, effectiveness, and understandability. All these are a mixture of TAM and DOI.

## 5. Results

This section presents the results based on the theoretical framework explained in Section 3 and methodology presented in Section 4.

### 5.1. Analysis of the existing solutions

According to the objectives of our analysis, six of the technological issues identified in Table 2 were analyzed to see if a relationship exists between technology adoption and technological issues and if one technological issue depends on another.

Based on the criteria described in Table 1, we critically analyzed the existing solutions. Table 3 presents details of the analysis showing the positive and negative aspects of the existing solutions.

**Table 3:** Analysis of the existing solutions.

**SN**	**Name of solution**	**Positive aspects**	**Negative aspects**
1	The HOLOBALANCE project	±	-, -*, –, 0
2	The My-AHA project	NA	NA
3	The HOPE project	+, +*, ++, ±	0
4	The Agnes project	+, ++, ±	-*, 0
5	The Pharaon project	+	-*, –, ≠, 0
6	The SMART-BEAR project	+, ±	-*, –, 0
7	The GATEKEEPER project	+	-*, –, ≠, 0
8	The SHAPES project	±	-, -*, –, 0
9	The FeelGood project	+	-* – ≠0
10	The MPOWER project	NA	NA
11	The OpenAAL project	+, ±	-*, –, 0
12	The PERSONA project	+	-*, –, ≠, 0
13	The RAFAALS project	+	-*, –, ≠, 0
14	Patent: An auxiliary system and a method for monitoring the health of the aged at home based on the IoT	+	-*, –, ≠, 0
15	Patent: Smart home service robot system based on diet and health management	+	-*, –, ≠, 0
16	Patent: Intelligent nursing home, intelligent management system	+	-*, –, ≠, 0
17	Patent: Cloud-computing-based method and apparatus for extraction and analysis of daily life and diet information of older adults living at home	+	-*, –, ≠, 0
18	Patent: Intelligent healthy diet recommendation system combined with mobile terminal	+	-*, –, ≠, 0
19	Patent: Monitoring system and sensor shoes for socials safety nets of older adults	+	-*, –, ≠, 0
20	Patent: Sports for older adults risk evaluation method	+	-*, –, ≠, 0
21	Patent: Intelligent exercise detection system based on multiple sensors and production device	+	-*, –, ≠, 0
22	Patent: Method for providing AI type care service for shopping healthcare company and game	+	-*, –, ≠, 0
23	Patent: IoT nighttime tracing light for older adults	+	-*, –, ≠, 0
24	Patent: Intelligent system for chaperoning of senior citizens	+	-*, –, ≠, 0
25	Patent: Mobile smart monitoring device for the apartments of older adults	+	-*, –, ≠, 0
26	Patent: Older adult care medical management system	+	-*, –, ≠, 0
27	Patent: Communication support robot system	+	-*, –, ≠, 0
28	The MobileAge project	+	-*, –, ≠, 0
29	The Homes4Life Certification project	+	-*, –, ≠, 0
30	The SmartHabits project: An Intelligent Privacy-Aware Home Care Assistance System	+	-*, –, ≠, 0
31	Active and Healthy Ageing at Work–A Qualitative Study with Employees 55–63 Years and Their Managers	NA	NA
32	I-CARE-SMART: co-creation in care for older adults	+	-*, –, ≠, 0
33	The SustAGE project	+	-*, –, ≠, 0
34	Intracom Medical ICT Solutions Portfolio	+	-*, –, ≠, 0
35	Joint deep learning and Internet of medical things based framework for older patients	+	-*, –, ≠, 0
36	Paper: Architecture and Implementation of an Internet Platform for Activating Older People: Case Study	+	-*, –, ≠, 0
37	Product: AIBO robot	+, +*, ++, ±	0
38	Product: NAO robot	+	-*, –, ≠, 0
39	Product: PARO robot	+, +*, ++, ±	0
40	Paper: ALL-VU system	+, ±	-*, –, 0
41	Paper: Senior App Suit	+, ++, ±	-*, 0
42	Paper: Virtual reality	+, ++, ±	-*, 0
43	Paper: Inferring loneliness levels in older adults from smartphones	+, ±	-*, –, 0
44	Paper: A Smart-Home System to Unobtrusively and Continuously Assess loneliness in Older Adults	+, ++, ±	-*, 0
45	The FACTAGE project—Fairer Active Ageing for Europe	NA	NA
46	Service: SeniAngel	+, +*, ++, ±, 1	NA
47	Patent: Old man dementia prevention and safety management system	+	-*, –, ≠, 0
48	Patent: Older adult health condition monitoring underwear based on somatosensory technology	+	-*, –, ≠, 0
49	Patent: Older people living alone monitoring device using the IoT	+	-*, –, ≠, 0
50	Patent: Device and method for acting as a friend in smart ageing service	+	-*, –, ≠, 0

### 5.2. Statistical evidence of analysis

From our initial discussion with selected respondents, we found out that they have a significant ability to use technology, especially operating their mobile devices and other digital devices they currently use, to adopt healthier lifestyles. The responses that we received are presented in Table 4.

**Table 4:** Responses from randomly selected people.

**Issue**	**P1%**	**P2%**	**P3%**	**P4%**	**P5%**	**P6%**	**P7%**	**P8%**	**P9%**	**P10%**	**AVE($\bar{X}$)**
Technological awareness	75	100	50	75	0	100	25	50	100	75	65
Support	75	75	25	100	50	75	25	50	50	75	60
Improve wellbeing	100	100	100	100	75	100	75	75	100	100	92.50
Safety and security	75	100	75	50	50	75	50	50	75	75	67.50
Ease of use	75	100	50	75	50	100	25	50	100	100	72.50
Privacy and confidentiality	75	75	50	75	50	100	25	50	75	75	65
Affordability	50	50	25	50	75	75	50	50	50	75	55
Confidence and trust	100	100	50	75	75	100	50	50	100	75	77.50
Willingness to accept technology	100	100	75	100	100	100	75	50	100	75	87.50
Satisfaction	100	100	75	75	75	100	75	50	100	75	82.5
Effectiveness	75	100	75	100	75	75	50	50	100	75	77.50
Understandability	75	100	75	75	75	100	75	50	100	100	82.50
Average ($\bar{X}$)	81.25	91.67	60.42	79.17	62.50	91.67	50	52.08	87.50	81.25	

SA = 100; PA = 75; N = 50; PD = 25; SD = 0; P Participant; $\bar{X}$ Average.

As Table 4 reflects, the response by each participant was captured for each issue that we identified as the major driver that positively affects technology adoption by older adults, based on the TAM and DOI theoretical frameworks.

We categorized the responses as “Yes,” “No,” and “Neutral.” “Yes” means that the respondent either strongly agreed or partially agreed that a given issue should be among the technology adoption issues to be considered. If the participant strongly agreed that a given issue should be included, 100% was assigned. If the participant partially agreed, 75% was assigned. If the participant's response was neutral and they neither agreed nor disagreed, 50% was assigned. “No” means that the respondent either partially disagreed or strongly disagreed on the matter. If the participant partially disagreed, 25% was assigned and if the participant strongly disagreed, 0% was assigned. The percentages reflecting the responses by the participants to the technology adoption issues are shown in Table 4.

**Figure 2:**
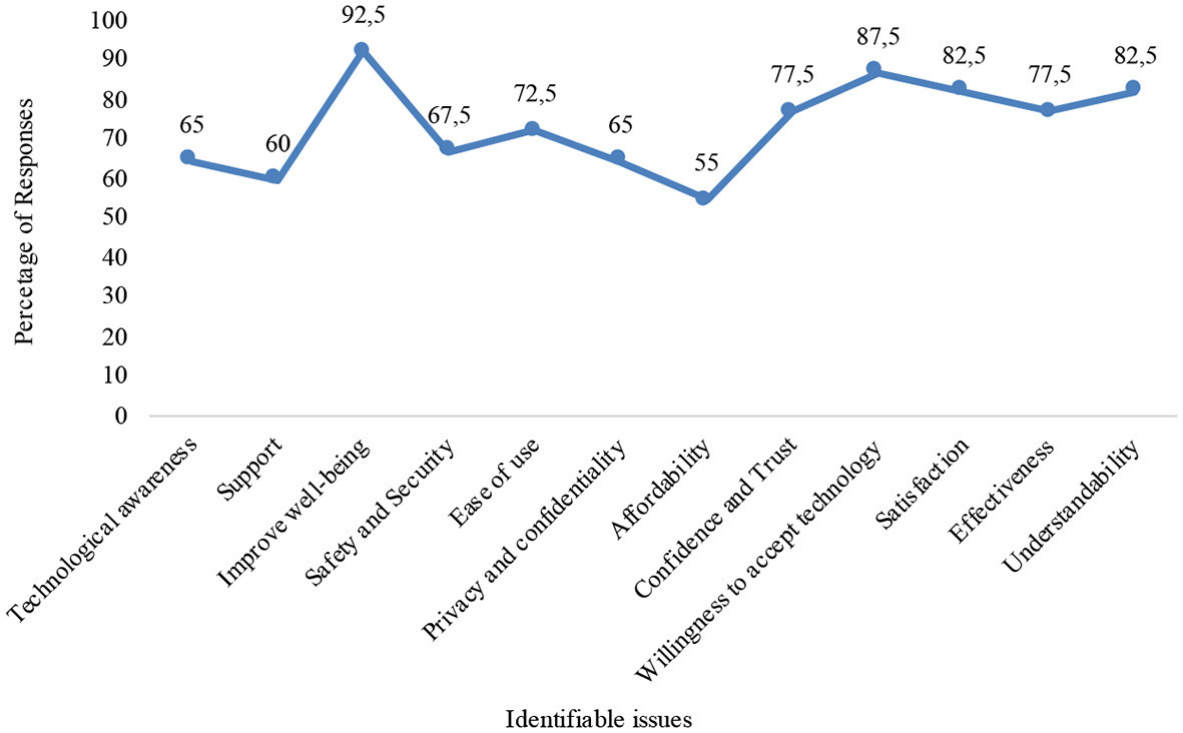
Technology adoption issues.

Additionally, we identified from the responses by the participants shown in Table 4 the degrees of their agreement about the importance of different technology adoption issues. The degrees of agreement are shown in Figure 2. According to the results, the technology adoption issue *improve wellbeing* has the highest relevance.

#### 5.2.1. *T*-test analysis

We first conducted a statistical test to check the validity of the following null hypotheses (*H*_0_) and alternative hypotheses (*H*_1_). Table 4 shows that the data is normal and meets the assumption for basic *t*-test analysis. The hypotheses were as follows:

#### I. Hypothesis on *improve wellbeing*

- *H*_0_*: Adopting technologies by older adults cannot improve their wellbeing*.
- *H*_1_*: Adopting technologies by older adults can improve their wellbeing*.

The input data: improve_wellbeing, *t* = 24.222, df = 9, *p*-value = 1.667e-09, 95% confidence interval: 83.86124–101.13876, sample estimates: mean of x = 92.5.

The average percentage of each response was calculated and the H0 test was applied to it. Adopting a sample *t*-test technique at ± = 0.05, the *t*- value of 24.222 was obtained. Using the standard *t*-table for 9 degrees of freedom, we reject the null hypothesis if the calculated value of *t* is greater than the critical value of *t*, which is 2.262. Since the obtained *t*-value of 24.222 is greater than the critical value of *t*, we reject the null hypothesis and accept the alternative hypothesis, which states that adopting technologies by older adults can improve their wellbeing.

#### II. Hypothesis on *technological awareness*

- *H*_0_*: Technological awareness is not statistically significant for technology adoption by older adults*.
- *H*_1_*: Technological awareness is statistically significant for technology adoption by older adults*.

The data input: technological_awareness, *t* = 6.0908, df = 9, *p*-value = 0.000181; 95% confidence interval: 40.85854–89.14146; Sample estimates: mean of x = 65.

The statistical test presented above shows that at the confidence interval of 95%, degree of freedom of 9, and alpha value of 0.05, the *t*-value is 6.0908, which is greater than the critical value of *t*, which is 2.262. Therefore, we reject the null hypothesis and accept the alternative hypothesis, which states that technological awareness is statistically significant for technology adoption by older adults.

#### III. Hypothesis on *willingness to accept technologies*

- *H*_0_*: Willingness to accept technologies is not statistically significant for technology adoption by older adults*.
- *H*_1_*: Willingness to accept technologies is statistically significant for technology adoption by older adults*.

The input data: willingness_to_accept_technology *t* = 15.652, df = 9, *p*-value = 7.79e-08; 95% confidence interval: 74.85416–100.14584; sample estimates: mean of x = 87.5.

The statistical test presented above shows that at the confidence interval of 95%, degree of freedom of 9, and alpha value of 0.05, the *t*-value is 15.652, which is greater than the critical value of *t*, which is 2.262. Therefore, we reject the null hypothesis and accept the alternative hypothesis, which states that willingness to accept technologies is statistically significant for technology adoption by older adults.

#### IV. Hypothesis on *understandability vs technology adoption*

- *H*_0_*: Understandability is not statistically significant for technology adoption by older adults*.
- *H*_1_*: Understandability is statistically significant for technology adoption by older adults*.

The input data: understandability; *t* = 15.461, df = 9, *p*-value = 8.67e-08; 95% confidence interval: 70.42927–94.57073; sample estimates: mean of x = 82.5.

Accordingly, at the confidence interval of 95%, alpha value of 0.05, and 9 degrees of freedom, the *t*-value is 15.652, which is greater than the critical value of *t*, which is 2.262. Therefore, we reject the null hypothesis and accept the alternative hypothesis, which states that understandability is statistically significant for technology adoption by older adults.

#### V. Hypothesis on *ease of use*

- *H*_0_*: Ease of use is not statistically significant for technology adoption by older adults*.
- *H*_1_*: Ease of use is statistically significant for technology adoption by older adults*.

The input data: ease_of_use; *t* = 8.3331, df = 9, *p*-value = 1.596e-05; 95% confidence interval: 52.81865–92.18135; sample estimates: mean of x = 72.5.

Accordingly, at the confidence interval of 95%, alpha value of 0.05, and 9 degrees of freedom, the *t*-value is 8.3331, which is greater than the critical value of *t*, which is 2.262. Therefore, we reject the null hypothesis and accept the alternative hypothesis stating that ease of use is statistically significant for technology adoption by older adults.

#### VI. Hypothesis on *support and technology adoption*

- *H*_0_*: Support is not statistically significant for technology adoption by older adults*.
- *H*_1_*: Support is statistically significant for technology adoption by older adults*.

The input data: support; *t* = 7.8558, df = 9, *p*-value = 2.559e-05; 95% confidence interval: 42.72249–77.27751; sample estimates: mean of x = 60.

Accordingly, at the confidence interval of 95%, alpha value of 0.05, and 9 degrees of freedom, the *t*-value is 7.8558, which is greater than the critical value of *t*, which is 2.262. Therefore, we reject the null hypothesis and accept the alternative hypothesis stating that support is statistically significant for technology adoption by older adults.

### 5.2.2. Correlation analysis

The main objective of this study is to understand the gap between the needs, provided solutions, and their adoption. We also identified several technology adoption issues and analysed their relevance. In this section, we analyze the correlation between several identified technology adoption issues by postulating a number of statistical hypotheses.

### I. Hypothesis on *technological awareness and willingness to accept technologies*

- *H*_0_*: There is no statistical relationship between technological awareness and willingness to accept technologies*.
- *H*_1_*: There is a statistical relationship between technological awareness and willingness to accept technologies*.

**Figure 3:**
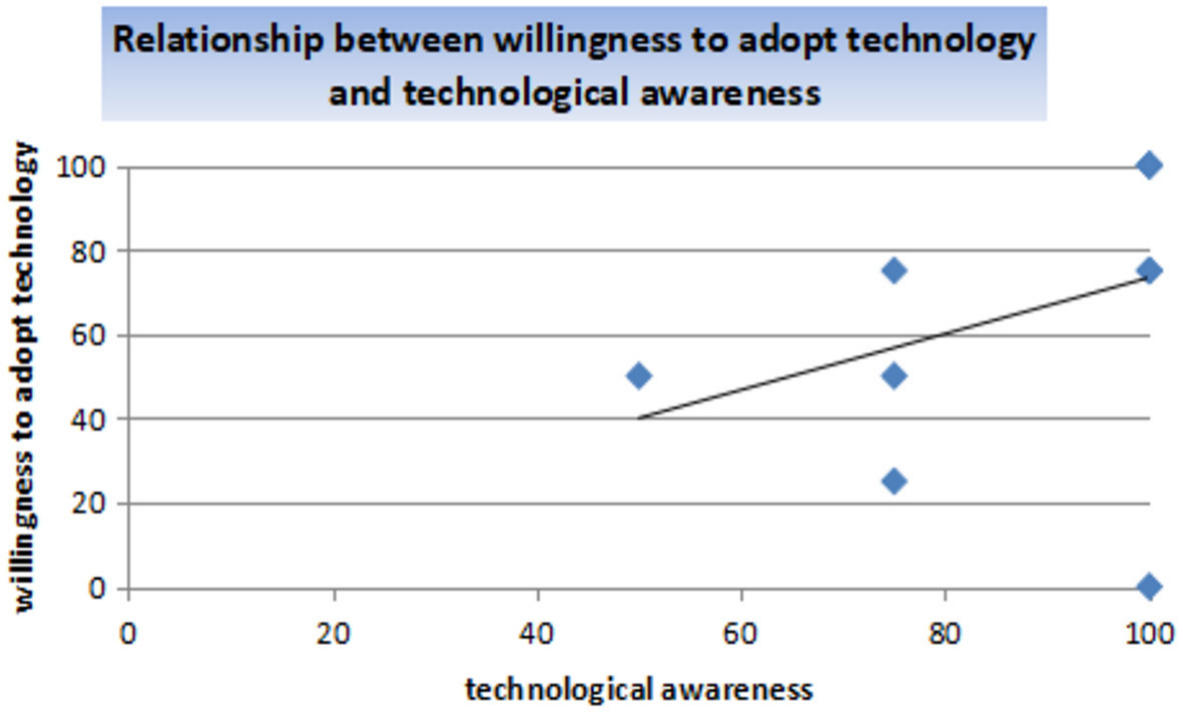
Correlation between technological awareness and willingness to accept technologies.

Figure 3 presents the analysis results of the correlation between technological awareness and willingness to accept technologies. The input data: technological_awareness and willingness_to_accept_technology; *t* = 1.0541, df = 8, *p*-value = 0.3226; 95% confidence interval: –0.3594439–0.8024112; sample estimates: correlation = 0.3492151.

The correlation between technological awareness and willingness to adopt technologies is 0.3492151, indicating a weak positive relationship between the variables. The *p*-value of 0.3226 indicates that the correlation coefficient is significant. However, there is a weak relationship between technological awareness and willingness to accept technologies, which may result from sentiments about the device or the inability to operate the device. The alternative hypothesis is accepted.

### II. Hypothesis on *support and improve wellbeing*

- *H*_0_*: There is no statistical relationship between support and improved wellbeing*.
- *H*_1_*: A statistical relationship exists between support and improved wellbeing*.

**Figure 4:**
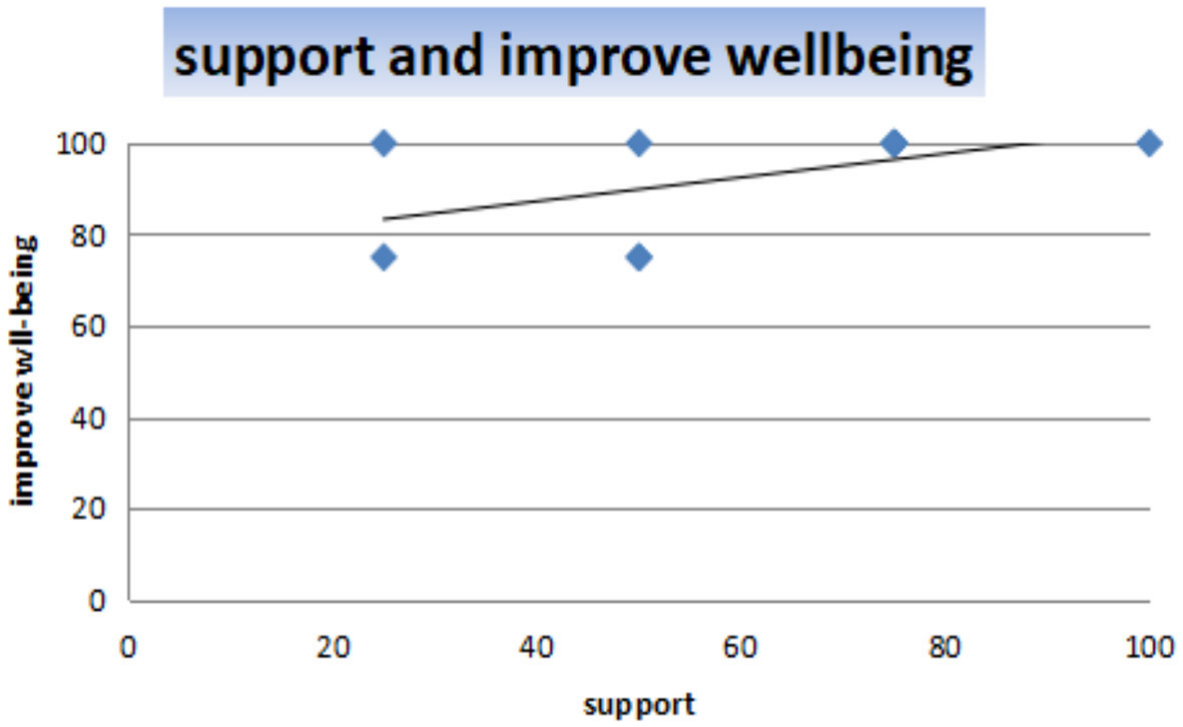
Correlation between support and improve wellbeing.

Figure 4 presents the analysis results of the correlation between support and improved wellbeing. The data input: support and improve_wellbeing; *t* = 1.7393, df = 8, *p*-value = 0.1202; 95% confidence interval: –0.1578894–0.8673726; sample estimates: correlation = 0.5238095.

The correlation between support and improve wellbeing is 0.5238095, which shows a moderate positive relationship between the variables, and the *p*-value of 0.1202 indicates that the relationship between support and improve wellbeing is statistically significant. Therefore, we can conclude that if the support obtained from technological devices increases, the wellbeing of older adults will improve.

### III. Hypothesis on *understandability and ease of use*

- *H*_0_*: There is no relationship between understandability and ease of use*.
- *H*_1_*: A relationship exist between understandability and ease of use*.

**Figure 5:**
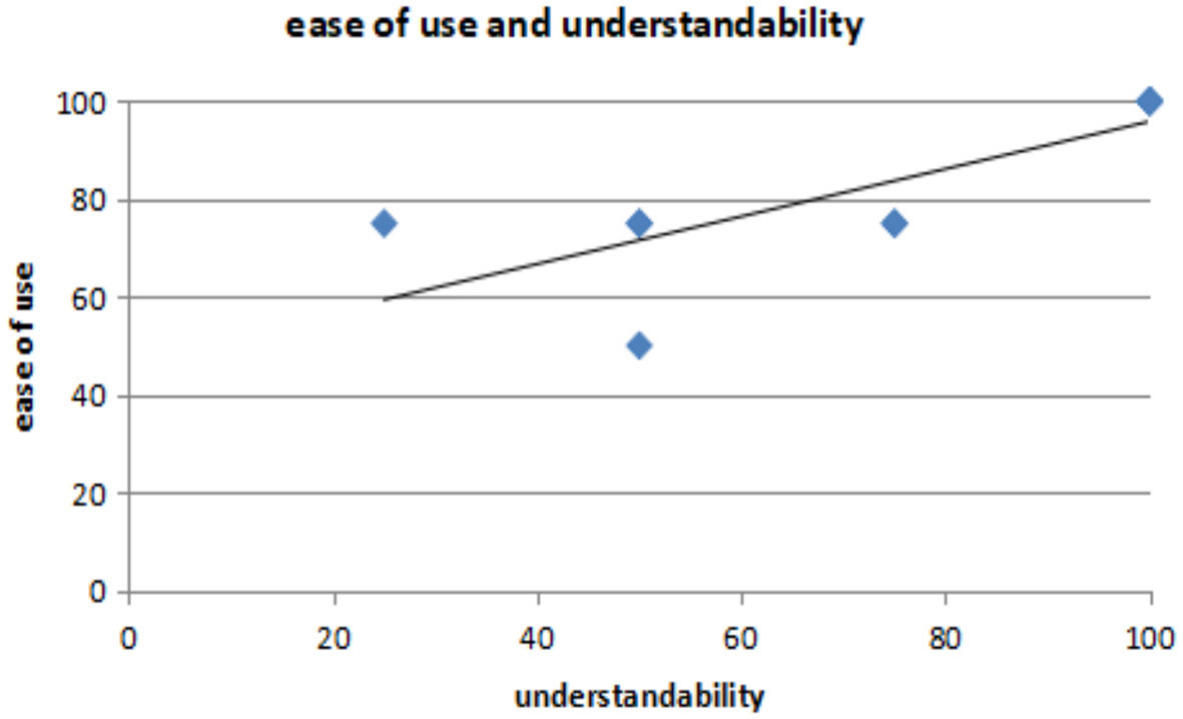
Correlation between understandability and ease of use.

Figure 5 presents the analysis results of the correlation between understandability and ease of use. The data input: understandability and ease_of_use; *t* = 3.6793, df = 8, *p*-value = 0.006225; 95% confidence interval: 0.3258361–0.9488141; sample estimates: correlation = 0.7928129.

The correlation between understandability and ease of use is 0.3492151, indicating a strong positive relationship between the variables. The *p*-value of 0.006225 also suggests that the correlation coefficient is significant. Therefore, a statistical relationship exists between understandability and ease of use.

## 6. Discussion

This section discusses the findings from selected existing solutions based on our results. Also, we discussed the implications of the statistical evidences of our analysis.

### 6.1. Some findings from selected existing solutions

This subsection further discusses some findings from solutions selected from Table 3.

Solution 1: The older adults found the system worked out in the HOLOBALANCE project encouraging and stimulating. The virtual coach was perceived as an alive, calm, intelligent, and friendly human. However, the usability of the entire virtual reality (VR) system showed a significant negative correlation with the participants' age. In the HOLOBALANCE project, mock-up interfaces were evaluated in semi-structured focus groups. Interviews were performed across three European countries. Also, a set of proof of concept validation studies were deployed, which aimed at assessing the accuracy of the different components of the sub-modules of the motion capture and assessment. The technology worked out in the project has potential for exploitation and commercialization as a service based on the IoT framework and on the accompanying business model of the continuous care and coaching platform. The validation results provide evidence that the proposed system can accurately support and assess physiotherapy exercises to care for balance disorders. This improves a patient's commitment to rehabilitation programs while enhancing the quality of the performed exercises. At the same time, we also indetified several negative aspects of the project in terms of technology adoption.

Solution 2: The My-AHA project is a typical ICT infrastructure with data analytics that is applied to the detection of frailty. The focus is not on technical innovation but on the concept of frailty and how to detect and take care of it. For this project, none of the criteria included by Table 1 were applicable.

Solution 3: The platform worked out in the HOPE project relies on a universal control box to interconnect a variety of devices. For this platform, the technology adoption involves buying the box and configuring the devices for it.

Solution 4: The Agnes project is an integration of ICT and social networking services that aids the detection of user states and activities and meeting the needs of older adults. The solution worked out in the Agnes project has the potential of prolonging for older adults the time spent at home, preserving health and promoting a healthy lifestyle, preventing social isolation, and providing support for (in)formal carers.

Solution 9: The FeelGood project provides a roadmap for an ecosystem for Finnish companies to excel in the international competition for services Personal Health Records (PHR). The PHRs form a part of the foundation of the healthcare system in Finland. The motivation of the project was to encourage the continuous utilization of e-services in the healthcare sector. The roadmap serves as a catalyst to transform the current illness-centered healthcare systems into a service landscape that allows patients and citizens to work in a partnership with healthcare providers to manage health issues. Several stakeholders participated in formulating a strategic roadmap for improving the PHR-based services in Finland. The roadmap focuses on providing an enabling environment for supporting and promoting healthcare concerns for the citizens through technology adoption. Although the formulated roadmap seems promising in terms of the approach and strategy employed, it is not certain whether the roadmap will be accepted by all the healthcare professionals and government.

Solution 10: The MPOWER project involves the cooperation platform, which is a technical integration platform of various services. It is a multisensor and multidevice environment that can provide support for the ageing population. However, technical solutions included by the platform are not directly applicable to senior users and their environments.

Solution 17: This solution systematically extracts and analyzes health and dietary information about older adults. It utilizes the information received from individual devices to form comprehensive and integrated information that can be used by different stakeholders, such as informal caregivers, to gain an insight into the QoL of older adults living at home.

Solution 18: This solution makes dietary recommendations and performs dietary monitoring of older adults. The recommendations are made based on the knowledge about the particular older adult rather than by following generic rules.

Solution 28: The MobileAge project produced the “Best Practice Guide for Co-creation of Open Public Services,” and is meant for co-design experts rather than end-users. The guide was evaluated in six co-creation case studies in Greece, Germany, the UK, and Spain but not in real-life cases. Unfortunately, the guide contains too many different methods without any clear directions as to in which order and situations one or another method should be applied. Furthermore, we could not find evidence of the OSCPSEP platform having been used in real-life case studies.

Solution 30: The SmartHabits project resulted in a system that was validated in a real environment, where a pilot application was set up in the city of Zagreb in cooperation with the foundation taking care of the older adults living alone. The validation confirmed that the proposed system has a potential to improve the quality of care by utilizing simple smart home sensors that can provide essential and continuous information about the occupant's status and environment. This was demonstrated in a scenario focused on prolonging the independence of the older adults living alone while offering peace of mind to their informal and formal caregivers. The system has great potential for adoption because of being non-invasive, self-adaptable to user behavior, zero-touch, and easy to manage. The most significant challenge of the adoption lies in the trust in technology.

Solution 32: The I-CARE-SMART project developed the following three methodological materials: ToolBox for Senior Engagement, ToolBox for Business Engagement, and Handbook on Co-Creation and Open Innovation Methods for Smart Care to Older Adults. These methods have not yet been validated in any real-life case studies.

Solution 33: The validation results of the pilots of the The SustAGE project have not been yet published. There is an extensive exploitation strategy, but its feasibility is hard to assess without knowing the validation results.

Solution 34: In the Intracom Medical ICT Solutions Portfolio project, a cloud-based integrated solution offering the PACS/RIS (Picture Archiving and Communication System / Radiology Information System) functionalities was provided as a service. Older adults are mentioned as potential customers, but there is no information about the actual usage or adoption.

Solution 35: In the “Joint deep learning and Internet of medical things based framework for elderly patients” project, the decision agent provides feedback to an inference engine of the target language analysis agent and the dialogue situation determination agent to allow for subjective interpretation of a given situation experienced by the older adult. However, devices that work and that do not require constant recharging remain challenges for this project.

Solution 36: In this research paper, an Internet platform for activating older adults was put forward. However, if an older person is unfamiliar with technologies, computers, and mobile devices, the platform is of no use. Therefore, a human assistant is required to get the platform started.

Solution 37: For the AIBO robot project, an off-the-shelf animal-like robot was provided to assist patients in hospitals where live animals are not allowed. In this case, a bond with the live animal is required. Additionally, the robot requires charging and has limited capabilities, but can still be beneficial.

### 6.2. Verifying the validity of statistical test

The validity test is 2-fold (see Section 5.2). First, the *t*-test indicates that the various technology adoption issues identified are statistically significant. For example, according to the statistical analysis, the *t*-value is bigger than the critical value of *t*, which is 2.262, at the confidence interval of 95%, degree of freedom of 9, and alpha value of 0.05. This means that technological awareness is statistically significant for older persons' embrace of technology. Moreover, the hypotheses postulated for statistical analysis of other technology adoption issues proved substantial based on the *t*-test. This means that the identified technology adoption issues should be considered when implementing suitable technologies for the aging population.

Secondly, the correlation analysis further clarifies the relationship between the technology adoption issues identified based on our formulated hypotheses. In addition, the correlation analysis provides evidence of the strength and course of action of the technology adoption issues identified. For example, our correlation analysis revealed a strong statistical relationship between understandability and ease of use based on the value 0.349215 and a *p*-value of 0.006225. However, we noticed a weak relationship between technological awareness and willingness (readiness) to accept technologies, possibly due to negative feelings about the technology hardware or an inability to operate it. Most profoundly, the correlation analysis revealed that support and improved wellbeing have a moderately positive correlation of 0.5238095, statistically significant at a *p*-value of 0.1202. This means that supports from Governments or organizations in terms of funding and other motivation will improve the wellbeing of the aging population.

### 6.3. Reflection and implication

Based on our analysis in Section 5.1, many technical solutions are still at the development stages. Because there is no clear implementation plan and funding, only a small percentage of these solutions are currently being used or are about to be used in real life. Therefore, the governments, providers, developers, etc., should be aware that when introducing a technology to older adults, the key drivers that facilitate its adoption should be considered. Also, they should be mindful that older adults may not have the necessary knowledge, skills, motivation, or confidence.

In Section 5.2, we used the quantitative approach to establish the technology's suitability for the ageing population. Regarding that, the null hypotheses and alternative hypotheses were formulated. We subjected these hypotheses to a statistical test to verify their validity. For that, the *T*-test and correlation analysis are used. While the *T*-test was used to statistically reveal the significant level of technology adoption issues, we used correlation analysis to understand the relationship between the several technology adoption problems that we identified.

The fundamental ways to improve technology adoption by older adults are (i) ensuring the motivational support in using these technologies (81, 82), (ii) creating an extensive awareness of its potential benefits through education and training exercises toward improving the QoL of older adults (83), (iii) ensuring a sustainable plan and measures toward incorporating these technologies into the lifestyles of older adults, (iv) working out a strategy or framework that ensures support by key organizations and their respective management for design and implementation of suitable technologies, (v) building reliable and trustworthy solutions within older adults' competencies to use the technologies, (vi) making the required facilities, such as Internet services, available, and (vii) involving all stakeholders, especially the older adults themselves in the requirements elicitation process to develop an acceptable technical solution (84). Significantly, a positive attitude toward adopting technologies by older adults can also positively influence their wellbeing.

## 7. Strengths and limitations

A strength of this study is that many existing technology solutions were analyzed. The analyzed solutions combine different projects, patents, and publications. This technology adoption review provides insight into the negative and positive features of the analyzed solutions for improving the QoL of older adults. Remarkably, the collaborative nature of the review process among the authors was instrumental to understand the current gaps between the needs, provided solutions, and their adoption from different perspectives.

Although the sample size of the respondents used for testing the hypotheses in Section 5.2 was small, still, there was diversity in the gender, age, ICT proficiency, and educational level of the older adults who answered the questions. In a small sample size, heterogeneity could be advantageous because it draws attention to critical features of the phenomena through a pattern across variance (85, 86). However, as part of our ongoing study, we intend to take into account bigger sample sizes across many nations, not just in Estonia for a more generalized and comparative results.

Another strength of our study is the combination of the TAM and DOI theoretical frameworks, which ensured that we caught pertinent viewpoints regarding analyzing the technology adoption concerns. A qualitative design was used, which made room for new viewpoints in identifying the strengths and weaknesses of each existing solution analyzed. Also, a quantitative method was used, enabling to test and formulate the hypotheses.

There are obviously some drawbacks in this study. First, the sample size used for testing the hypotheses is rather small, which has a detrimental impact on the generalizability of the results. Additionally, the older adults' prevailing circumstances and state of mind when answering the questions may have influenced their narratives and response. This leaves place for future work since a large scale study would bring further benefit to the research community.

## 8. Conclusions and recommendations

This paper investigated the technological solutions from the technology adoption perspective based on the TAM and DOI technology adoption frameworks using the mixed method research approach. First, our analysis reveals both the positive and negative aspects of using technologies by older adults to improve their QoL. Second, we used a statistical metric to establish the appropriateness of our analysis further. Thirdly, we made an essential contribution based on our in-depth analysis by providing crucial recommendations and policy implications for consideration below in this section.

Consequently, we recommend, as a policy, the full support of governments and private organizations to design and implement holistic solutions. In our opinion, the involvement of governments in driving the campaigns for adopting technologies toward increasing the QoL of older adults is inevitable for successful technology adoption. Support by a government can be implemented in terms of funding and enacting laws that give relevance and attention to the ageing population of a society.

Additionally, we recommend that privacy concerns of older adults should be further considered based on, for example, the framework suggested in Khan and Gambo (87), when implementing any technical solution for improving QoL and wellbeing.

## Author contributions

IG, KT, KM, RZ-G, AA, MM, MB-D, FM, and AL: conceptualization and writing—review and editing. IG and KT: methodology and data analysis. IG, KT, KM, RZ-G, AA, MM, and AL: formal analysis and investigation. KT: resources, supervision, and funding acquisition. IG: writing—original draft preparation. KT and KM: project administration. All authors have read and agreed to the published version of the manuscript.

## Footnotes

^1^https://bit.ly/3RmsS3W.
